# *Nymphaea lotus* Linn. (Nymphaeaceae) Alleviates Sexual Disability in L-NAME Hypertensive Male Rats

**DOI:** 10.1155/2019/8619283

**Published:** 2019-07-29

**Authors:** Poumeni Mireille Kameni, Djomeni Paul Desire Dzeufiet, Danielle Claude Bilanda, Marguerite Francine Mballa, Ngadena Yolande Sandrine Mengue, Tchinda Huguette Tchoupou, Agnes Carolle Ouafo, Madeleine Chantal Ngoungoure, Theophile Dimo, Pierre Kamtchouing

**Affiliations:** ^1^Laboratory of Animal Physiology, Faculty of Science, University of Yaounde I, P.O. BOX 812, Yaounde, Cameroon; ^2^Laboratory of Biological Sciences, Faculty of Science, University of Bamenda, P.O. BOX 39 Bambili, Bamenda, Cameroon

## Abstract

Hypertension (HT) is a risk factor for erectile dysfunction (ED). This study aimed to evaluate the suppressive effect of* Nymphaea lotus* (*N. lotus*) on erectile dysfunction induced by NO deficiency in rat. 40 male rats equally divided into 4 groups received an oral treatment with 10 mg/kg/day of L-NAME, a NO blocker, during 4 weeks. Control group composed of 10 male rats received only distilled water (10 mL/kg). Thereafter oral treatments with* N. lotus* (75 and 200 mg/kg/day) and losartan (10 mg/kg/day) started and continued concomitantly with L-NAME in 3 groups for 4 additional weeks. Normal and negative controls received only distilled water. Sexual behaviour, orientation activities, anxiety, and penile histomorphology were evaluated at the end of treatment. L-NAME administration elevated significantly the blood pressure in male rats and decreased the copulatory rate by enhancing intromission latency and decreasing the numbers of intromission and ejaculation. However, the sexual motivation remains unaltered by chronic NO blockage suggesting that L-NAME induces penile dysfunction mainly by peripheral mechanisms. L-NAME chronic intake also induced anxiety, 4 weeks of* N. lotus* cotreatment prevented inhibitory effects of L-NAME on male sexual behaviour by shortening mainly ejaculation latency and postejaculatory interval while losartan does not. Losartan proved to be a more effective drug to decrease the blood pressure compared to the plant extract. Effectively,* Nymphea lotus* was able to reverse totally at 75 mg/kg the increment of hemodynamic parameters and the histological damage and exhibit anxiolytic-like effects in hypertensive male rats.* Nymphaea lotus* uses NO pathway to facilitate sexual responses at central and peripheral levels and can have a double medicinal use, against anxiety and erectile dysfunction.

## 1. Introduction

Erectile dysfunction (ED) is defined as the inability to achieve and/or maintain sufficient erection to allow satisfactory sexual intercourse. This symptom is quite common in people suffering from hypertension (HT). In fact, 30% of them present ED compared to normotensive people. The severity of this secondary condition is directly proportional to the severity of HT [1, 2]. Effectively, the evolution of HT is associated with several deleterious effects on the structure and function of the systemic blood vessels, so penile vascularization is not spared. The HT gradually deteriorates the integrity of the endothelial tissues, which are the support of the erection mechanism. The dysfunction of these tissues thus promotes the gradual installation of ED and is continually maintained by oxidative stress and inflammatory conditions associated with HT [3]. Indeed, free radicals and oxidative stress are toxic to the endothelium. They interfere with the signaling pathway of nitric oxide (NO), causing damage responsible for the occurrence of ED [4].

Other causes responsible for ED in people with HT are, namely, a deficiency in NO or even a psychological or nervous condition [5]. However, the involvement of the nervous system has not been clarified in the ED related to HT.

Currently, plants have received more attention regarding their use as therapeutic agents. In Cameroon,* Nymphaea lotus* Linn (*N. lotus*)—a plant belonging to the family Nymphaeaceae—is used in popular medicine for its aphrodisiac, astringent, and anti-inflammatory properties [6]. It would also produce sedative effects on the nervous system [7, 8]. Besides, pharmacological long-term blockage of NO synthesis by the chronic administration of L-NAME, an inhibitor of nitric oxide synthase (NOS), has been reported to produce systemic arterial hypertension, vascular structural change, and erectile dysfunction in animal models [9, 10]. Thus the present work has been undertaken to assess the effects of* Nymphaea lotus *Linn flowers on peripheral and central components of the mating behaviour in a rat model of NO deficiency-induced hypertension.

## 2. Materials and Methods

### 2.1. Preparation of Plant Extract

*Nymphaea lotus* Linn flowers have been collected and prepared as previously described [11]. The flowers were cleaned, dried in the shade, and then crushed. The powder obtained served for the preparation of the aqueous extract (50 g of powder per liter of tap water). The dry extract was then reconstituted in distilled water at appropriate concentrations for the experiment and administered orally in a volume of 10 mL/kg body weight.

### 2.2. Preliminary Qualitative Phytochemical Analysis

According to the secondary compounds highlighted, the following reagents were used for phytochemical analysis of the aqueous extract of* N. lotus* flowers: Flavonoids (Mg^2+^), alkaloids (modified Dragendorff reagents), saponins (frothing test), Tannins (FeCl3), reducing substances (Fehling A and B Solutions), cardiac glycosides (Salkowski test), phenols (FeCl_3_ and K_3_Fe (CN)), anthocyanins (acid), and lipids (filter paper) [12].

### 2.3. Quantitative Phytochemical Analysis

The Folin-Ciocalteu'method [13] was used for the determination of total phenolic content which was calculated using a calibration curve of gallic acid serial dilutions and expressed as mg of gallic acid equivalents per g of dried extract. The total flavonoid content was determined using a calibration curve of the rutin and expressed in mg of rutin per g of dry extract [14].

### 2.4. Drugs and Chemicals

N*ω*-nitro-L-arginine methyl ester (L-NAME) and urethane were obtained from Sigma-Aldrich (St. Louis, MO, USA). Estradiol benzoate was purchased from Sigma Chemical company. Progesterone caproate was procured from Bayern Schering Pharma (Berlin, Germany) and losartan (Losar-Denk 50) was obtained from Denk Pharma (München, Germany).

### 2.5. Animal Treatment

The experiments were carried out in accordance with the principles of laboratory animal protection approved by the Ethics Committee of the University of Yaoundé I. Male Wistar rats used for experimentation were provided by the animal house of the Animal Physiology Laboratory (University of Yaoundé I, Cameroon). These eight weeks old rats were sexually experimented by cohabitation with females (ratio 2:1), under a natural day light cycle, and supplied* ad libitum* with water and soy free chow. After 4 weeks of cohabitation, a pretest was carried out and animals displaying hyposexual activities (< 1 ejaculation on average) were disqualified for the study. The others were randomly divided into five groups of 10 animals each and treated according to their respective body weights.

  Group I: rats receiving only distilled water;
  Group II: L-NAME-treated rats (10 mg/kg);
  Group III: rats treated with L-NAME + losartan (10 mg/kg);
  Group IV: rats treated with L-NAME + extract of* N. lotus* (75 mg/kg);
  Group V: rats treated with L-NAME + extract of* N. lotus* (200 mg/kg).

**Figure 1:**
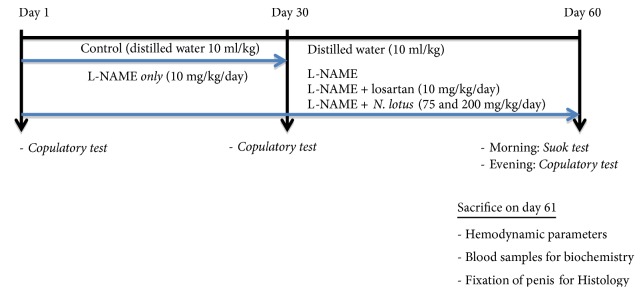
Experimental procedure.

The animals received L-NAME alone for 4 weeks, and then the different treatments were added concomitantly to the latter (Figure 1). All these products have been administered orally. After the last administration (Day 60), rats were fasted all night; blood pressure and heart rate were recorded in the morning (Day 61).

### 2.6. Behavioural Tests

#### 2.6.1. Measurement of Anxiety and Motor-Related Behaviour

The rat Suok test (ST) is based on the measurement of animal behaviour ethological analysis of the animal's exploration in the elevated novel alley following a protocol described by Kalueff et al. [15]* (Supplementary Material).*

#### 2.6.2. Measurement of General Mating Behaviour

The experiment was carried out on day 60, one hour after the final treatment of the male rats. The experiment was conducted at 7:00 pm under dim light. Receptive female rats (estradiol benzoate 12 *μ*g in olive oil injected intramuscularly 48 h prior to pairing plus progesterone 0.5 mg in olive oil injected intramuscularly 6 h prior to pairing) were introduced into the cages of male animals with 1 female to 1 male. The observation for mating behaviour was immediately commenced and continued for 30 minutes according to the method used by Kada et al. [16]. The following measurements were recorded or calculated: mount latency, the time from onset of the test to the first mount with or without penile insertion; intromission latency, the time from the introduction of the female to first penile insertion; ejaculatory latency, time from the first intromission to ejaculation; mount number, the number of the mounts without intromission prior to ejaculation; intromission number, the number of mounts with intromission before ejaculation; postejaculatory interval, time from ejaculation to the first intromission of the second copulatory series; copulatory efficiency, a measure of intromission success (calculated as percentage of mounts in which the male gained vaginal insertion).

Temporal patterning was expressed as non-“postejaculation” pauses number, the number of pauses following mount or intromission interrupted before ejaculation, and the cumulative duration of the non-“postejaculation” pauses, the summary of interval from the beginning of one pause to the start of the next mount.

The test was terminated if the male failed to evince sexual interest after 10 minutes from the beginning of observation. If the female did not show receptivity, another artificially warmed female replaced it.

### 2.7. Biochemical Parameters

#### 2.7.1. Blood Pressure and Heart Rate Measurements

Blood pressure and heart rate were recorded by direct method [17]. Rats were anaesthetized with intraperitoneal injection of 15% ethyl carbamate (1.5 g/kg). The trachea was exposed and cannulated to facilitate spontaneous respiration. A polyethylene catheter was inserted into the rat carotid artery. This catheter was linked to the transducer connected to the recorder hemodynamic Biopac Student Lab MP type 35. Another catheter was inserted into the femoral vein and a bolus injection of 10% heparin (0.1 mL/100g body weight) was immediately administered. The animal was then equilibrated for at least 15 min before the blood parameters (systolic and diastolic blood pressure) and heart rate was recorded.

#### 2.7.2. Relative Penile Weight and Histological Analysis

After recording of cardiovascular parameters, the animals were sacrificed by decapitation. Thereafter penis was removed and weighed using 4-digital electronic balance (Mettler PL301). For histological studies, the penile tissues were fixed in 4%PFA for 48 hours and then dehydrated in graded (50-100%) alcohol and embedded in paraffin. Thin sections (5 *μ*m) were cut with a microtome (Reichert-Jung 2030) and stained with Mallory Trichrome staining for observations.

### 2.8. Data Analysis

Statistical analysis was carried out using the Statistical Package for Social Sciences version 21.0 for MAC (SPSS Inc, Chicago, IL, USA) software. Data are presented as mean ± standard error of mean (S.E.M.). One-way analysis of variance (ANOVA), followed by Tukey post hoc test, was used. A probability level less than 0.05 was accepted as significant.

## 3. Results

### 3.1. Preliminary Qualitative Phytochemical Analysis of the Aqueous Extract of* N. lotus *Flowers

The aqueous extract of* N. lotus* contained flavonoids, alkaloids, saponins, tannins, anthraquinones, phenolic compounds, glycosides, cardiac glycosides, but not anthocyanins and lipids (Table 1).

### 3.2. Total Phenolic and Flavonoids Contents of* N. lotus*

In* N. lotus* aqueous extract, total phenolic compound was found to be 71.29±0.11 mg/g of dried extract, calculated as gallic acid equivalent and total flavonoids compound was 7.15±0.87 mg/g of dried extract, calculated as rutin equivalent.

### 3.3. Effect of* N. lotus* Aqueous Extract on General Features of the Experimental Animal Groups

The L-NAME treatment did not alter body weight and penile weight significantly. In the same manner, losartan or* N. lotus* did not alter these parameters.

The systolic, mean, and diastolic blood pressures were significantly higher in the L-NAME group compared to the control group. Effectively, the chronic L-NAME (10 mg/kg) treatment significantly increased mean blood pressure (MBP) (P<0.001) versus baseline whereas additional treatment with* N. lotus* (75 mg/kg) and losartan (10 mg/kg) prevented the increment of arterial blood pressure. Contrarily,* N. lotus* at the level of 200 mg/kg significantly (P<0.001) enhanced the mean blood pressure compared to baseline (Table 2).

### 3.4. Effect of* N. lotus* Aqueous Extract on Behaviour

#### 3.4.1. Effect of* N. lotus* Aqueous Extract on Anxiety and Motor-Related Behaviour

L-NAME administration during 60 days induced a reduction of directed exploration (34.38%), horizontal (HA) and vertical activities respectively by 19.91% and 45.45% compared to control, whereas it provokes an significant increase (P<0.001) of latency to leave (LL), as well as a significant increase of missteps (MS) (P<0.01) and motor incoordination index (MI) (P<0.01). Cotreatments with losartan and plant extract at the doses of 75 and 200 mg/kg induced respectively a decrease of the LL by 25.84%, 36.48%, and 43.91% compared to nontreated animals; by 26.55%, 38.98%, and 13.56% of the number of HA; and by 29.58%, 43.64%, and 56.01% of the number of MS and MI compared to L-NAME group. Contrarily to losartan, additional treatment with* N. lotus* at both doses induced a significant decrease (P<0.05 and P<0.01) of the number of defecation compared to control. All general features of the animal groups are presented at Table 3.

#### 3.4.2. Effect of* N. lotus* Aqueous Extract on Orientation Activities

**Figure 2:**
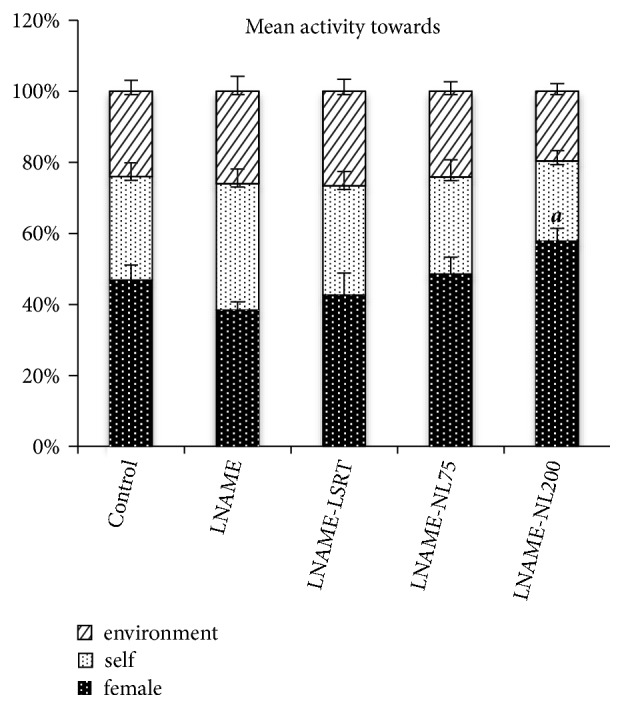
Effect of aqueous extract of* Nymphaea lotus *flowers on orientation activities in L-NAME treated male rats. Each bar represents the mean ± S.E.M. of group;  ^a^ is significantly different compared to control. The exact value of P is mentioned in the text.

The aqueous extract of* Nymphaea lotus *flowers at the dose level of 200 mg/kg markedly influenced the orientation behaviour of the hypertensive animals, which showed more attraction towards female rats (Figure 2).

#### 3.4.3. Effects of Hypertension on Male Rat Sexual Behaviour

After 60 days, L-NAME treatment drastically reduced the number of animals displaying ejaculation. Effectively all the animals achieved mounting (Table 4), but only 4/10 rats treated with L-NAME reached ejaculation within 30 min of testing (Table 5).

#### 3.4.4. Effect of* N. lotus* Aqueous Extract on the Inhibition of Sexual Behaviour Induced by L-NAME

The administration of L-NAME for 60 days to male rats resulted in distress in the sexual vigor of male rats, as evidenced by parameters of sexual motivation and copulatory performances studied (Tables 4 and 5). 30 days of cotreatment with losartan restored mount and intromission frequency in normal range whereas* N. lotus* reduced significantly (P<0.05) the frequency of mount compared to control. Both doses reduced mount frequency in a significant manner (P<0.001) compared to losartan (Figure 4, Table 4).

Meanwhile the intromission latency decreased significantly (P<0.01) following cotreatment with the plant extract at the dose of 75 mg/kg (P<0.05) and 200 mg/kg (P<0.001) compared to L-NAME. The reference drug, losartan, did not modify intromission latency compared to hypertensive untreated animals. The ejaculatory latency was absent as no ejaculation was observed in the group cotreated with losartan (Table 5).

#### 3.4.5. Effects of Chronic Treatment with L-NAME on Temporal Patterning

**Figure 3:**
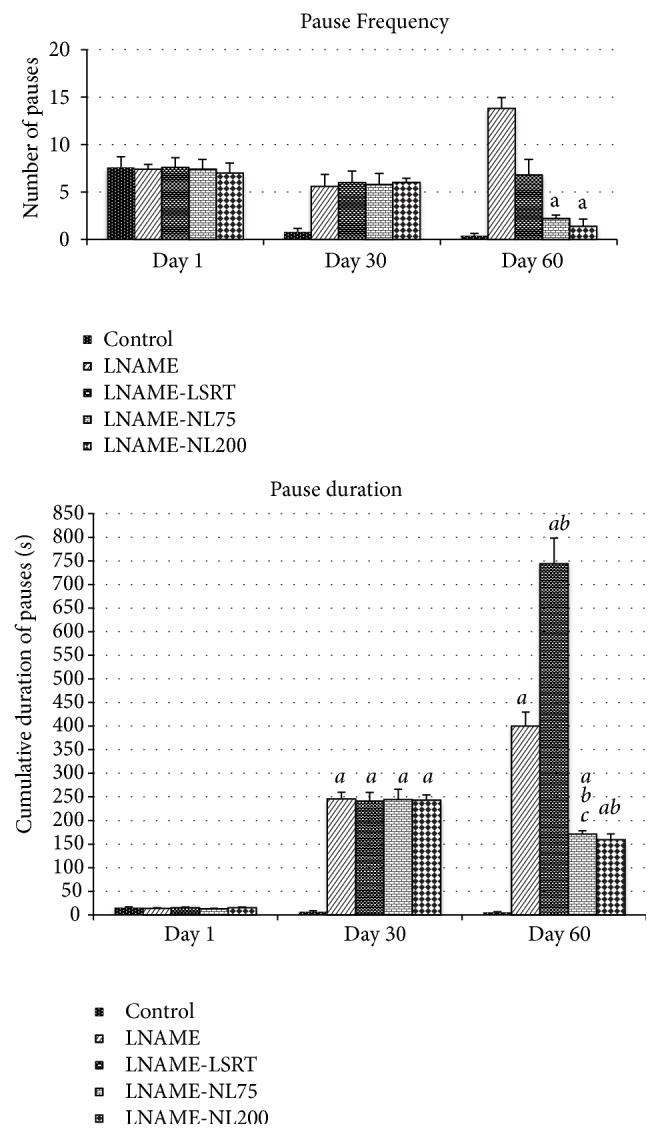
Effects of chronic treatment with L-NAME (10 mg/kg/day), losartan (10 mg/kg/day) or* N. lotus *(75 and 200 mg/kg/day) on temporal patterning of sexual behaviour of male rats.

The effects of L-NAME on temporal patterning sexual behaviour of male rats are presented in Figure 3. L-NAME treatment increased the* non-*“*postejaculation”* pauses number and their cumulative duration (P<0.001). 30 days cotreatment with the plant extract induced a considerable decrease in the number of pauses (P<0.001) and on their cumulative duration in relation to control group.

### 3.5. Histology

**Figure 4:**
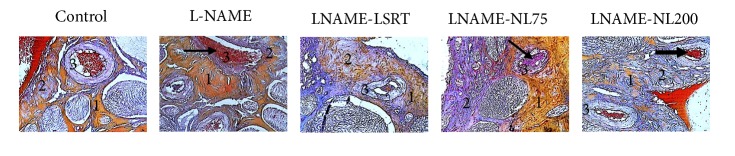
Effects of* N. lotus* on penile histomorphology of hypertensive rats. Penile cross sections. X200; tissue sections were stained with Mallory. (1) Smooth muscle cells (orange), (2) collagen (blue), and (3) penile arteries. Arrows indicate vascular congestions.

Mallory Trichrome staining revealed in L-NAME treated rat, vascular congestion (Figure 4), a decrease in smooth muscle proportion (red) and an increase in collagen level (blue) in penile tissue.

## 4. Discussion

The present results provide, for the first time, scientific information concerning the ability of* Nymphaea lotus* flowers aqueous extract to improve sexual performances and alleviates anxiety in L-NAME hypertensive rats. Our previous studies have demonstrated that treating rats with L-NAME causes injury to the vascular endothelium [11], and this model is widely used to study hypertension, as well as its relative complications [18]. The results obtained in this study showed that chronic NO synthesis inhibition with L-NAME treatment at the level of 10 mg/kg for 8 weeks, caused a significant increase in blood pressure and a nonsignificant variation of heart rate compared to control. Long-term administration of the L-arginine analogue (L-NAME) to normotensive rats can induce NO-deficient hypertension. The precise mechanism is based on the fact that NO is synthesized and released from endothelial cells to mediate vasorelaxation and L-NAME reduces NO production resulting in increased total peripheral resistance and high blood pressure [19, 20]. These effects were significantly prevented by treatment with* N. lotus* at the dose of 75 mg/kg. Several explanations are possible for the constancy in heart rate of hypertensive male rats. One possibility is that the baroreceptor reflex is (indirectly) reset as a consequence of the constant chronic increase in blood pressure. Surprisingly, our results revealed an exacerbation of the blood pressure increment in rat cotreated with the extract of* N. lotus* at the dose of 200 mg/kg, suggesting dose-dependent modulatory properties of our plant extract on blood pressure.

Mount, intromission, and ejaculation frequencies are useful indices of vigor, libido, and potency [21]. An increase in ML reflects sexual motivation and increase in the number of IF and EF shows the efficiency of erection and the ease by which ejaculatory reflexes are activated [16]. After 30 days of L-NAME administration, we observed in this study a significant deficit in intromission and ejaculatory frequencies with the low efficiency of copulatory behaviour whereas mount latency and frequency remain unaltered by the L-NAME treatment compared to control. These parameters were exacerbated after additional 30 days of L-NAME treatment, suggesting progressive deficit in general consummatory mechanisms. Further, the reduced mean copulatory interval and the low percentage of copulatory efficiency exhibited in hypertensive animals indicated that it was not every mount that resulted in intromission, suggesting an erectile dysfunction in hypertensive male rats. Other authors also showed that sexual dysfunctions such as decreased libido, delayed orgasm, difficulties in maintaining an erection, and inhibition of ejaculation are common side effects of NO deficiency [9, 22].

Sexual response arousal component reflected by the mount latency appears to be not affected by the L-NAME treatment. The temporal patterning of sexual behaviour analysis probes the most complex sociosexual behaviour [10]. In a normal sexual behaviour pattern of male rat, a pause occurs only for the refractory period after an ejaculation or after satiation. Surprisingly, in this study, we observed a significant progressive increase in the number and latency of pauses not preceded by ejaculation in animals treated with L-NAME. Besides, the increased number of attempts (mounts), the prolonged refractory periods (postejaculatory interval), and finally the reduction of the copulatory efficiency suggest a difficulty to penile insertion or a general fatigue that could be explained by the pathologic increment of the blood pressure. Cotreatment with the aqueous extract of* N. lotus* flowers restores partially or totally the deficits provoked by L-NAME treatment on consummatory patterns.

Altogether, our results indicate that the aqueous extract of* N. lotus* flowers increases both sexual potency and motivation. The appetitive component of the sexual behaviour in rats is usually related to the variations of intromission latency and postejaculatory intervals, but the executive counterpart is estimated to be proportional to the changes observed in the number of mount or intromission, the ejaculation latency, and the mean copulatory interval [23]. Compounds derived from plants, such as flavonoids, have been reported to directly affect male sexual functions [24] by increasing vasorelaxation of cavernosum smooth muscle cells through activating NO-cGMP pathway [25], or interacting with central pathways that participate in libido or sexual arousal [26].

Sexual function in hypertensive rats was found to be decreased and could be due to decrease in smooth muscle level and increase in collagen level of penile tissue as evident from histopathological study. Hypertension progression is associated with progressive vascular structural damage [27] and, as our data suggest, penile vascularization does not remain unaffected. Described remodelling effects of L-NAME-induced hypertension have been effectively prevented only by the aqueous extract of* N. lotus* flowers at the dose of 75 mg/kg while losartan did not have a remarkable effect compared to model, mainly at the penile vessels level. It is mentioned that drugs that improve endothelial function, antihypertensive* like* ARA (losartan), are unable to ameliorate erectile function since they do not possess specificity of action at the level of the penile vasculature and smooth muscle cells [28]. Besides, the plant extract exhibited a localised protective potential that protects the penile vasculature from structural damage, supporting positive impact on penile blood flow, and therefore on the penile erectile function.

Chronic blockage of NO by L-NAME also produced more displacement grooming and motor coordination deficits as assessed by increased missteps in this study, suggesting anxiety and reduced locomotor capacity in hypertensive animals [16]. Other authors previously reported that treatment with L-NAME may induce anxiety [29] and locomotor deficiency [30]. Cotreatment with the aqueous flowers extract of* Nymphaea lotus* at the both doses produced an anxiolytic-like effect as evidenced by the delayed latency to leave the center of the elevated alley (suok test), the reduced defecation and motor incoordination episodes compared to hypertensive nontreated animals. Similar to other Nymphaea genus species, our results also show that* Nymphaea lotus *possess anxiolytic properties [6, 31]. The prosexual and anxiolytic effects of* N. lotus* were consistently observed in several measures but principally in the group given the lowest dose of aqueous extract.

Considering the high comorbidity of anxiety with sexual disorders in men [32] or those with cardiovascular diseases, the anxiolytic-like effect produced by* N. lotus* at a dose with prosexual and antihypertensive effects could be considered as an additional benefit of the plant extract. Thus,* Nymphaea lotus* could be considered as a potential dual medicinal agent to treat anxiety and erectile dysfunction.

## 5. Conclusion

*Nymphaea lotus* is able to modulate blood pressure, to restore erectile function by interacting with both central and peripheral pathways. We thus suggest that the aqueous extract obtained from this plant could be considered to facilitate sexual responses regulated by both the NO pathway at central and peripheral levels.

## Figures and Tables

**Table 1:** Phytochemical screening of aqueous flowers extract of *Nymphaea lotus* Linn.

Tests	Results
Tannins (Ferric chloride test)	+

Phenolic compounds (FeCl3 and K3Fe (CN))	+

Reducing substances (Fehling A and B test)	+

Alkaloids (Dragendorff's test)	+

Anthocyans (Acid)	-

Cardiac glycosides (Salkowski test)	+

Flavonoids (Mg^2+^)	+

Lipids (Filter paper)	-

Saponins (Frothing test)	+

(+): present and (-): absent.

**Table 2:** Effect of *Nymphaea lotus *on general features of the experimental animal groups after 60 days of treatment.

Groups	Body Weight (g)	Penile Weight (g)	PBR (×10^−2^)	Heart rate (bpm)	Blood pressure (mmHg)		
					Systolic	Mean	Diastolic
Control	195.20±12.65	0.29±0.02	15.05±0.67	337.82±4.67	97.87±2.69	88.52±1.96	83.85±3.15

L-NAME	212.80±7.47	0.28±0.01	13.28±0.38	371.40±3.38	154.39±6.29 **a**	147.73±4.09 **a**	144.40±3.17 **a**

LNAME-LSRT	200.80±11.93	0.29±0.01	14.66±1.06	354.38±13.73	103.95±4.77 **b**	101.40±4.00 **b**	100.13±5.00 **b**

LNAME-NL75	217.00±7.64	0.32±0.02	14.61±1.13	360.13±3.42	134.27±11.08 **ac**	131.53±1.54 **abc**	130.16±4.94 **ac**

LNAME-NL200	212.00±8.67	0.29±0.01	13.47±0.18	380.55±11.34 **a**	157.70±3.79 **ac∗**	151.14±4.44 **ac∗**	149.37±4.80 **ac**

Each value represents the mean ± SEM of group. For comparisons among control and experimental groups, a = significantly different compared to Control; b = significantly different compared to L-NAME group; c = significantly different compared to LNAME-LSRT; *∗* dose-dependence between two extract doses. The exact value of P is mentioned in the text. PBR: penile weight/body weight. SBP: systolic blood pressure, DBP: diastolic blood pressure, MBP: mean blood pressure, HR: heart rate.

**Table 3:** Effect of *N. lotus* on anxiety and ambulatory parameters.

Measures and behavioural domains		Mean ± SEM				
		Control	LNAME	LNAME-LSRT	LNAME-NL75	LNAME-NL200
(I) Exploration						

HA	Horizontal activity	44.20±1.36	35.40±2.64	26.00±1.67 ***a***	21.60±2.01 **a**	30.60±2.29

VA	Vertical activity	4.40±0.68	2.40±0.75	2.60±0.40	4.80±0.73	4.60±0.68

SA	Stopping activity	3.00±0.32	1.40±0.24	1.60±0.40	1.80±0.58	2.80±0.58

DE	Directed exploration	32.00±2.24	21.00±1.22	23.80±4.04	28.20±1.59	34.00±1.73

LL	Latency to leave	8.87±0.73	35.64±1.52 **a**	26.43±2.86 **ab**	22.64±2.98 **ab**	19.99±1.29 **ab**

ID	Interstop distance	15.50±1.99	28.30±4.96	19.93±4.13	15.95±3.65	12.45±1.99 **b**

(II) Displacement (D)						

FD	Frequency	3.40±0.24	2.40±0.24	5.40±0.68	2.00±0.32	3.40±0.51

DD	Duration (s)	16.00±0.71	12.20±1.74	31.00±3.11	17.80±0.73	23.80±2.91

(III) Vegetative behaviours						

DB	Defecation	4.00±0.32	3.00±0.45	4.60±0.24	0.60±0.40*** ac***	0.00±0.00*** abc***

UR	Urination	0.4±0.4	0.00±0.00	1.00±0.63	0.20±0.20	0.00±0.00

(IV) Motor coordination						

NF	Falls	0.00±0.00	0.00±0.00	0.00±0.00	0.00±0.00	0.00±0.00

MS	Missteps	1.80±0.49	5.80±0.58*** a***	4.60±0.81 ***a***	4.40±0.51 ***a***	5.00±0.95 ***a***

MI	Motor incoordination index	1.80±0.49	5.80±0.58*** a***	4.60±0.81 ***a***	4.40±0.51 ***a***	5.00±0.95 ***a***

Each value represents the mean ± SEM of group. For comparisons among control and experimental groups, a = significantly different compared to Control; b = significantly different compared to L-NAME group; and c = significantly different compared to LNAME-LSRT. The exact value of P is mentioned in the text.

**Table 4:** Effect of *N. lotus* on consummatory patterns of hypertensive male rats.

Parameters	Days of treatment	Consummatory parameters				
		Control	LNAME	LNAME-LSRT	LNAME-NL75	LNAME-NL200
Mount frequency	Day 1	31.60±2.87	44.80±1.46 ***a***	44.80±3.25 ***a***	44.40±1.96 ***a***	42.20±1.39 ***a***
	Day 30	46.20±3.40	63.60±2.04 ***a***	66.40±2.11 ***a***	65.60±1.29 ***a***	64.20±2.06 ***a***
	Day 60	61.40±3.37	53.20±3.87	68.00±2.11	43.80±4.89 ***ac***	47.40±3.56*** c***

Intromission frequency	Day 1	30.40±2.62	40.20±3.69	41.00±3.92	38.00±0.71	40.40±1.86
	Day 30	44.60±3.53	60.40±1.21 ***a***	62.80±1.46 ***a***	62.00±1.73 ***a***	60.40±2.25 ***a***
	Day 60	59.60±4.38	30.60±2.44 ***a***	42.80±6.63	43.40±5.18	45.00±3.39

Ejaculation frequency	Day 1	2.60±0.24	1.20±0.58	1.20±0.37	1.20±0.37	1.00±0.45
	Day 30	2.80±0.37	0.60±0.40 ***a***	0.60±0.40 ***a***	0.60±0.24 ***a***	0.60±0.40 ***a***
	Day 60	3.20±0.20	0.20±0.20*** a***	0.00±0.00	1.60±0.51 ***ac***	2.00±0.55 ***bc***

Penile licking	Day 1	30.00±2.28	40.80±4.02	42.60±3.17	40.00±1.14	41.80±3.48
	Day 30	36.00±4.48	42.40±4.50	46.40±6.44	42.80±7.22	51.80±6.46
	Day 60	59.60±4.38	30.40±1.94*** a***	41.40±5.90	42.00±5.21	44.60±3.20

Mean copulatory interval (sec)	Day 1	1152.27±72.14	908.16±30.44***a***	893.56±51.68***a***	902.48±43.63***a***	914.23±42.80***a***
	Day 30	1370.25±66.88	919.55±46.17 ***a***	548.78±30.12 ***ab***	565.51±39.71 ***ab***	575.28±33.85 ***ab***
	Day 60	1347.91±37.90	582.19±55.25*** a***	Absent	1433.39±51.87 ***b***	1467.93±30.83 ***b***

Copulatory efficiency (%)	Day 1	96.58±2.62	89.24±5.91	91.99±8.20	86.31±4.29	95.65±2.32
	Day 30	96.42±1.61	95.18±1.98	94.76±2.20	94.46±1.09	94.06±1.42
	Day 60	96.61±2.23	58.59±5.87 ***a***	61.88±7.11*** a***	98.67±1.33*** bc***	95.02±2.68 ***bc***

Each value represents the mean ± SEM of group. For comparisons among control and experimental groups, a = significantly different compared to Control; b = significantly different compared to L-NAME group; and c = significantly different compared to LNAME-LSRT. The exact value of P is mentioned in the text.

**Table 5:** Effects of *N. lotus* on different parameters of sexual motivation of hypertensive male rats.

Copulatory parameters	Days of treatment	Parameters of sexual motivation				
		Control	LNAME	LNAME-LSRT	LNAME-NL75	LNAME-NL200
Mount latency (sec)	Day 1	26.60±2.09	25.60±2.01	23.80±1.16	25.20±1.62	25.60±0.87
	Day 30	20.40±1.17	25.40±1.08	24.20±1.59	26.60±1.33 ***a***	24.80±1.36
	Day 60	13.80±0.84	17.55±1.96	14.08±1.48	9.09±1.07*** b***	8.60±1.05 ***b***

Intromission latency (sec)	Day 1	30.60±1.60	63.80±2.44 ***a***	64.80±1.46 ***a***	62.80±4.69 ***a***	60.00±2.41 ***a***
	Day 30	22.60±0.60	49.20±2.75 ***a***	48.00±1.76 ***a***	43.40±2.50 ***a***	37.80±1.56 ***abc***
	Day 60	14.20±0.71	33.90±4.10 ***a***	32.37±1.72 ***a***	23.12±2.50 ***b***	14.60±1.32*** bc***

Ejaculation latency (sec)	Day 1	520.22±21.82	636.14±25.82	656.31±48.61	634.43±65.27	631.74±38.43
	Day 30	345.30±33.78	484.14±16.88 ***a***	482.89±22.48 ***a***	473.83±21.75 ***a***	465.74±36.81 ***a***
	Day 60	295.99±17.95	664.04±22.65 ***a***	Absent	277.59±13.35 ***b***	269.26±19.06 ***b***

Postejaculatory interval (sec)	Day 1	419.00±29.83	425.20±22.68	389.00±16.82	409.20±17.36	430.00±13.68
	Day 30	294.68±15.16	641.84±15.12 ***a***	649.00±8.53 ***a***	641.20±13.79 a	645.44±16.72 ***a***
	Day 60	252.74±14.48	714.48±18.72 ***a***	Absent	315.26±12.21 ***ab***	285.61±14.44*** b***

Each value represents the mean ± SEM of group. For comparisons among control and experimental groups, a = significantly different compared to Control; b = significantly different compared to L-NAME group; and c = significantly different compared to LNAME-LSRT. The exact value of P is mentioned in the text.

## Data Availability

The datasets analysed during the current study are available from the corresponding author upon reasonable request.
